# Role of Glycosylation in Vascular Calcification

**DOI:** 10.3390/ijms22189829

**Published:** 2021-09-11

**Authors:** Ainun Nizar Masbuchin, Mohammad Saifur Rohman, Ping-Yen Liu

**Affiliations:** 1 Institute of Clinical Medicine, College of Medicine, National Cheng Kung University, Tainan 70457, Taiwan; nizar.fkub08@gmail.com; 2 Department of Cardiology and Vascular Medicine, Faculty of Medicine, Universitas Brawijaya, Malang 65111, Indonesia; ippoenk@ub.ac.id; 3 Division of Cardiology, Internal Medicine, National Cheng Kung University Hospital, College of Medicine, National Cheng Kung University, Tainan 70403, Taiwan

**Keywords:** vascular calcification, medial calcification, O-glycosylation, N-glycosylation, proteoglycan

## Abstract

Glycosylation is an important step in post-translational protein modification. Altered glycosylation results in an abnormality that causes diseases such as malignancy and cardiovascular diseases. Recent emerging evidence highlights the importance of glycosylation in vascular calcification. Two major types of glycosylation, N-glycosylation and O-glycosylation, are involved in vascular calcification. Other glycosylation mechanisms, which polymerize the glycosaminoglycan (GAG) chain onto protein, resulting in proteoglycan (PG), also have an impact on vascular calcification. This paper discusses the role of glycosylation in vascular calcification.

## 1. Introduction

Vascular calcification (VC) is an independent predictor of mortality and morbidity in cardiovascular disease [1]. Coronary artery plaque calcification increases mortality 1.7-fold, whereas an extensive form increases it 60-fold [2]. It also increases economic burden in the form of cardiovascular disease [3]. Vascular calcification is an active process of depositing calcium in the form of hydroxyapatite in the vascular matrix [4]. 

Currently, vascular calcification is considered to resemble bone ossification and it has pro- and anti-calcification factors. An imbalance of these factors in certain diseases such as atherosclerosis, chronic kidney disease (CKD), and diabetes lead to vascular calcification formation [5]. These factors are regular proteins that need canonical post-translational modification (PTM) processing, and the most common PTM is glycosylation. Some of these factors will undergo glycosylation to become fully functional. Accumulating evidence highlights the importance of glycosylation in vascular calcification. In this review, we summarize and discuss the latest evidence of glycosylation in vascular calcification.

## 2. Classification, Clinical Consequences, and Risk Factors of Vascular Calcification

Vascular calcification is an ectopic mineralization and can be classified into two forms depending on where the mineral is deposited [6,7]. The first type is intimal calcification, which is commonly observed in atherosclerosis, hyperlipidemia, and metabolic syndrome [8]. It is a patchy and discontinuous process involving macrophage and vascular smooth muscle cells (VSMC) in the lipid-rich region of atherosclerotic plaques [9]. The second type of VC is medial calcification, which is commonly observed in CKD, diabetes mellitus, aortic aneurysm, and aging patients. This type of VC (called Monckeberg’s sclerosis) is described as sheet-like calcification in the medial layer with concentric thickening of the vessel wall [9,10], and it is less directly correlated with inflammation [4,11]. 

Both of the calcification types have their own clinical consequences. The clinical complications of intimal calcification are plaque rupture, myocardial infarction, and stroke. Medial calcification increases arterial stiffness, arterial pulse pressure, increased pulse wave velocity, and all-cause mortality [8]. Both of the VC types share common and specialized mechanisms. Common mechanisms include oxidative stress, apoptosis, mitochondrial dysfunction, mechanical stress, and cell death. Loss of inhibitors and VSMC senescence are special drivers of medial calcification, whereas the inflammation process is found in intimal calcification [8]. The risk factors for developing intimal calcification are advanced age, diabetes mellitus, dyslipidemia, hypertension, being male, smoking, and hyperphosphatemia. Advanced age, diabetes mellitus, renal dysfunction (decrease in glomerulus filtration rates), hypercalcemia, hyperphosphatemia, and dialysis duration are risk factors for developing medial calcification [12]. 

## 3. Mechanism of Vascular Calcification

Bone is largely composed of hydroxyapatite and hydroxyapatite crystal grown from an extruded matrix vesicle (MV). Matrix vesicle buds from osteoblasts. It serves as the nidus for calcium-phosphate deposition within the vascular wall [13,14]. Vascular smooth muscle cell can similarly exude MVs as a mechanism to promote VC [4]. Matrix vesicle contains enzymes and factors including alkaline phosphatase. They concentrate calcium and initiate hydroxyapatite mineral crystallization. The matrix vesicle in the vasculature is analogue to the MV of skeletal tissue. VSMC, like osteoblasts, can also produce nucleating vesicles as a result of primary osteogenic differentiation [15].

Vascular calcification is considered an active process. Four different VC factors have long been proposed. They are: (1) loss of inhibition, (2) induction of bone formation, (3) circulating nucleation complexes, and (4) cell death [16]. A passive process is involved in this process via a thermodynamic mechanism, which is elevated calcium and phosphate-induced apatite nucleation and crystal growth. The induction of bone formation results from the differentiation of pericytes and/or VSMCs. Circulating a nucleating complex or locally released MV serves as a site for calcium crystallization. Dead cells also serve as nucleating sites for calcium crystals [11]. 

Physiologically, VC is inhibited by the balance of inhibitors and inducers. The inhibitors are required to prevent vascular calcification in basal conditions. At present, VC inhibitors are identified. They are inorganic pyrophosphatase (PPi), matrix Gla protein (MGP), osteopontin (OPN), osteoprotegerin (OPG), fetuin-A, and klotho [17]. The calcification inducers include phosphate, bone morphogenic protein 2 (BMP2), alkaline phosphatase (ALP), and fibroblast growth factor 23 (FGF23) [5]. 

Vascular calcification is initiated by MVs released by apoptotic cells and proliferative-phenotype VSMCs [11]. These MVs are deposited in the vessel wall and serve as a nucleation site for calcification [13,18]. Such vesicles contain proteins related to calcification and enzymes that are responsible for extracellular matrix cross-linking [13]. Under high-phosphate conditions, VSMC uptakes phosphate via a phosphate transporter, Pit-1. VSMC will eventually undergo differentiation to osteoblast-like cells and subsequently downregulate calcification inhibitors [7].

## 4. Regulatory Factors in Vascular Calcification

### 4.1. Calcification Inhibitors

Normally, healthy contractile-phenotype VSMC secretes MV containing MGP and Fetuin-A to inhibit calcification [14]. MGP is a well-established inhibitor of vascular calcification. MGP can inhibit BMP as well as calcium mineral itself. MGP requires post-translational modification to be active and needs vitamin K dependent carboxylation. Epidemiological studies support the role of vitamin K in vascular calcification. Warfarin, which is capable of inhibiting vitamin K, can inhibit MGP carboxylation. Therefore, vitamin K deficiency supports vascular calcification [15].

Fetuin-A, a protein secreted by hepatocyte into circulation, is capable of inhibiting ectopic calcification in a CKD model [19,20]. Fetuin A exerts its inhibitory activity by forming colloid with minerals called calciprotein particles (CPP) [21]. It only inhibits de novo calcium phosphate formation [22]. Fetuin-A is endocytosed by VSMC in the presence of high calcium [23] and it reduces the ability of MV secreted by endocytosing VSMC to calcify [18]. It seems that the ability of fetuin-A in decreasing VC is concentration-driven. Low levels of fetuin-A decrease VC, whereas high levels increase inflammation, thereby increasing cardiovascular events. Recent studies revealed that a higher level of fetuin-A increases risk of cardiovascular mortality in patients with moderate calcium scores and exacerbates the progression of cardiac dysfunction [24,25].

The receptor activator of nuclear factor-κB ligand (RANKL), which is a factor for regulating osteoclastic activity, can induce VC via the osteogenic differentiation factor RUNX2. Oxidized lipids and oxidants can increase RANKL in VSMC. RANKL has a soluble receptor called OPG. OPG treatment reduces vascular calcification. The effects of OPG treatment may occur via downstream pathways, MSX2, and ALP [15]. BMP2 plays an important role in vascular calcification in atherosclerotic plaques, CKD, and diabetes mellitus [10,26].

Inorganic pyrophosphatase (PPi) can strongly inhibit hydroxyapatite formation in VC [5]. PPi is circulated and locally secreted by VSMCs. Bellosta et al. [27] demonstrated that PPi secreted by VSMC is important in decreasing vascular calcification.

Recently, autophagy became more of a concern regarding VC. Autophagy is the mechanism of internal cellular components’ digestion that recycles nutrients and energy. It is involved in survival, differentiation, and functioning bone cells [28]. Evidence has shown that autophagy decreases VC [29,30].

### 4.2. Calcification Inducer

Phosphate can induce mineralization physico-chemically. It also regulates and coordinates cell signaling through a specialized transportation that promotes calcification. The Pit-1 is a channel that is responsible for phosphate co-transport into VSMC. The increasing cellular phosphate level signals osteogenic gene expression and suppresses VSMC genes. β-glycerophosphate is a well-known inducer of VSMC calcification and is commonly used for studying VSMC calcification in vitro. The Pit-1 is subject for regulation by calcium, BMP2, and platelet-derived growth factors (PDGF). Increasing these factors can upregulate Pit-1 [5].

BMP2, BMP4, and BMP6 increase plaque formation via pro-inflammatory and pro atherogenic effects and promote differentiation. BMP2 increases RUNX2, the master transcriptional regulator to promote mineralization, in VSMC. BMP2 and BMP4 are also associated with VC in CKD. High phosphate (which is commonly observed in CKD) and BMP2 induce mineralization in addition to RUNX2 and MSX2 expression in a β-catenin-dependent manner. BMP2-MSX2 signaling promotes calcification in diabetic vascular disease by inducing myofibroblast to osteoblast-like cells [22]. BMP2 also regulates Pit-1, which is a phosphate transporter. The increasing BMP leads to an increase in Pit-1 and subsequently leads phosphate transport into cells, thereby promoting VC [5].

Transforming Growth Factor β (TGFβ) is a member of the TGFβ family, which belongs to the TGFβ superfamily. The BMP family also belongs to the TGFβ superfamily [31]. In general, the binding of TGFβ to its receptor activates smad 2/3 as downstream signaling, whereas BMP ligand binding to its receptor leads to smad 1/5/8 signaling [31]. TGFβ can also exert its effects through a non-smad pathway (through the Mitogen Activated Protein Kinase (MAPK) pathway). TGFβ-1 is known to have the ability to induce VSMC calcification by stimulating it to differentiate into osteoblast-like cells [32]. It is also highly expressed in calcified aortic valve tissue [33]. However, Shimokado et al. reported the paradox that TGFβ decreases VSMC calcification [34]. This result can be explained as follows: activation smad2/3 by TGFβ inhibits RUNX2 expression, leading to a decrease in calcification, whereas the activation of non-smad pathways by TGFβ yields the opposite effect. In contrast to TGFβ, smad 1/5/8 activation by BMP2 increases RUNX2 levels and activity, leading to calcification [35]. Therefore, the effect of TGFβ in VC is likely dependent on the activated downstream pathway. Activating a non-smad pathway (MAPK pathway) could lead to RUNX2 activation and subsequently induce VC [35]. Furthermore, TGFβ is involved in the induction of MGP, which acts as a vascular calcification inhibitor [36]. Finally, evidence showed complex cross-talk between TGFβ, BMP, and other pathways [35], which has implications for decreasing VC [37].

The alkaline phosphatase is an enzyme that breaks down the mineralization inhibitor pyrophosphatase. Additional novel phosphatase is found in a matrix vesicle called phosphatase, orphan 1 (PHOSPHO1). Inhibition of PHOSPHO1 in VSMC also inhibits matrix calcification in vitro [15]. Recent evidence reported that glycosylation differences contribute to catalytic properties among bone alkaline phosphatases [38].

FGF23 is the most potent phosphate regulator. FGF23 is synthesized by osteocytes and secreted into circulation. It exerts its effects after binding to a receptor, klotho, in the kidneys. This binding allows the regulation of phosphate absorption [39]. FGF23 is reported to be expressed in human calcified vascular tissue [40].

Cellular components are also involved in the calcification process. The VSMC has long been identified as a cell that is capable of differentiating into osteoblast-like cells through various mechanisms [41,42]. Resident pericytes have the ability to transdifferentiate into bone-forming cells as well [17,43,44]. Bone-marrow-derived cells also contribute to chondrocyte-like cells in intimal calcification [45].

## 5. Glycosylation

Glycosylation is the post-translational process of the enzymatic addition of a saccharide compound to another saccharide, protein, or lipid [46,47]. Protein glycosylation pathways take place in the secretory pathways (endoplasmic reticulum (ER) and golgi), cytoplasm, nucleus, and mitochondria [48]. Glycosylation marks protein for further processing, e.g., tags protein for insertion into the cell membrane or secretion from the cell. It also modifies intracellular proteins [49]. Glycosylation helps protein to fold properly and can inhibit protein degradation by protecting it from proteases [50,51,52]. Glycosylation is involved in a wide variety of biological processes, such as receptor interaction, in addition to protein secretion and transport. A protein can have multiple glycosylation sites [53]. Glycosylation is a multiple-step process involving many enzymes. The stages of glycosylation consist of initiation, core extension, branch elongation, and capping [48]. There are 16 distinct glycosylation pathways, which are distinguished by sugar-protein linkage, initial monosaccharides linked to protein, and unique initiating enzymes [48], including N-glycosylation, O-glycosylation, C-manosylation, phosphoglycosylation, and glypiation [54]. 

N-linked glycosylation and O-linked glycosylation are the major types of glycosylation found in mammalian cells [55,56,57]. N-linked glycosylation is the attachment of glucose to an arginine residue. N-linked glycosylation takes place in ER and golgi [58]. The initiation process of this glycosylation takes place in ER co-translationally or post-translationally, which involves the oligosaccharyltransferase complex (OST) [48]. O-glycosylation is the addition of a glucose compound to the Thr/Ser residue of a target protein. Eighty percent of secreted proteins that traffic secretory pathways are O-glycosylated [57]. In contrast to N-linked glycosylation, O-linked glycosylation takes place post-translationally only in golgi [59,60]. Enzymes involved in this glycosylation are involved in the initiation process. Initiation of O-linked glycosylation is catalyzed by the GALNTs family and it has functional redundancy [61]. Some complex glycans (e.g., proteoglycans) are composed of either N-linked or O-linked glycosylation through a process of initiation and elongation [62]. The initiation step is the formation of a tetrasaccharide linker, catalyzed by different enzymes: the UDP-xyl transferase, UDP-Gal transferase I, UDP-Gal transferase II, UDP-Glc transferase I, and UDP-Gal transferase, respectively [62]. The fifth saccharide in the linker will determine the fate of the glycosaminoglycan (GAG): whether it will become a chondroitin/dermatan sulfate GAG or heparan sulfate/heparin GAG. Elongation of a linear chain is catalyzed by different enzymes depending on the fate of the GAG. In chondroitin sulfate, for example, the elongation of the linear repeating disaccharide region is catalyzed by a chondroitin polymerase complex, which consists of many enzymes [63]. Common GAG found in human tissues include chondroitin sulfate, dermatan sulfate, heparan sulfate, and heparin [62]. 

Glycosylation is involved in many diseases [64]. It has been the main research focus regarding cancer for decades [46,65]. It is also thought to contribute to the development of diabetic nephropathy by interfering with kidney tubular cells’ ion transport, leading to necrotic cell death [66]. Glycosylation is also involved in cardiovascular diseases [67]. The recent genome-wide association study (GWAS) revealed a correlation between glycosylation and lipid metabolism, including HDL and triglyceride, which has been extensively studied [68,69]. Glycosylation is also involved in the development of atherosclerosis disease [70]. GWAS revealed that the gene responsible for glycosylation is associated with coronary in-stent restenosis (ISR) [71]. Adhikara et al. [72] highlight the importance of GAG synthesis in the development of atherosclerosis. Finally, accumulating evidence supports the role of glycosylation in calcification and bone metabolism [73,74] as well as chondrogenic differentiation [75]. Recent studies also reported on the role of glycosylation in VC [76]. The glycosylation of VC regulatory factors leads to various effects on VC (Table 1).

Besides the enzymatic glycosylation explained above, there is also non-enzymatic glycosylation, which does not require enzymes. It is a reaction that leads to the formation of an advanced glycation end product (AGE), which is a commonly-observed non-enzymatic glycosylation product in diabetic patients [48]. This review focuses on enzymatic glycosylation.

## 6. N-glycosylation and Vascular Calcification

N-glycosylation is the most widely studied glycosylation. The work of Siddals et al. [76] was the first study to link N-glycosylation with VC. As many epidemiological studies found a correlation between statin and VC, Siddals et al. used statin to deplete the substrate required for N-glycosylation and also used tunicamycin as a specific N-glycosylation inhibitor. In the presence of β-Glycerophosphate as a calcification inducer, they found that statin and N-glycosylation inhibitors attenuate inhibitory effects of IGF in VC. They also found that N-glycosylation inhibitors decrease IGFR surface expression and its downstream signaling.

BMP2 is a well-known signaling molecule involved in VC [10,26]. It promotes phosphate uptake, phenotypic modulation, and the calcification of human VSMCs [88]. It has been reported that BMP has N-glycosylation and O-glycosylation sites [89]. Hang et al. [77] reported that N-glycosylation is required for BMP2 secretion and osteoblast differentiation. Mutation of an N-glycosylation site at N135Q impairs BMP-2 secretion. Overexpressing the mutant causes accumulation and induces ER stress. N-glycosylation is required for the proper folding of BMP2 proteins. The mutation also reduces BMP2’s ability to induce osteoblast differentiation. Furthermore, van de Watering et al. reported that non-glycosylated BMP2 has reduced biological activity compared to glycosylated BMP2. However, their study found that non-glycosylated BMP2 retains its ability to induce osteoblastic differentiation [90]. Other groups worked on BMP2 receptors and found that N-glycosylation of BMP2 receptors enhance the binding to its ligand, BMP2 [91]. It is known that the BMP2 receptor type 2 bears an N-glycosylation site in the extracellular domain [92]. Despite this evidence, there are currently no reports of a direct correlation between BMP2 glycosylation and vascular calcification. The role of BMP2 and its receptor in inducing VC, however, is well known. Therefore, it is tempting to hypothesize that modifying the glycosylation on this receptor could possibly affect VC.

TGFβ has been identified as a cause of vascular calcification by promoting VSMC differentiation to osteoblast-like cells [32,93]. TGFβ is subjected to N-glycosylation [94]. Treatment with tunicamycin (an inhibitor of N-glycosylation) blocks TGFβ secretion from the cell, and therefore leads to intracellular accumulation. Moreover, its receptor, TGFβR, is also subjected to N-acetylglucosamine (GlcNAc) regulation. TGFβR is glycosylated in the extracellular domain, which is the binding domain. Removal of glycosylation impairs interaction with its ligand and fails to elicit downstream signaling [95]. Indeed, blocking glycosylation affects vascular calcification. Wen et al. [78] demonstrated that blocking fucosylation, the specialized form of N-glycosylation, decreases vascular calcification. The absence of fucose core in TGFβR markedly dysregulates downstream TGFβ/smad2/3 signaling.

Fetuin-A harbors two N-glycosylation and two O-glycosylation sites in the A chain and two O-glycosylation sites in the B chain [96]. Fetuin-A is well-known as anti-vascular calcification [22]. Evidence has shown that phosphorylated fetuin-A is associated with aortic stiffness and renders pro-calcification milieu [97]. A genetic study revealed the association of polymorphism of the fetuin gene (ASHG) and calcium serum [98], and, surprisingly, the risk of VC [99]. Furthermore, mutation analysis by another group found that *AHSG* gene mutation causes a change in glycosylation. This mutation changes Thr to Ser at position 256 and eventually decreases fetuin-A O-glycosylation. The same study also revealed the increase in fucosylation on the N-glycosylation site Asn-176 in sepsis patients [100]. Although there have been no studies that directly correlate fetuin-A glycosylation and VC, the results from the genetic association studies and glycoproteomic studies seem convincing and, therefore, need to be further elaborated.

Galectin-3 is a member of the β-galactoside-binding lectin family, which interacts with β-galactoside on glycoconjugate expressed on cell surface receptors and cellular matrix proteins. It has been implicated in the control of cell differentiation, growth, adhesion, migration, and apoptosis [101,102]. It is expressed in various tissues, resides in the cytoplasm or nucleus, and is also secreted out from the cell [103]. Epidemiological studies showed a correlation between galectin-3 and aortic calcification [104]. Recently, galectin-3 is involved in vascular osteogenesis by inducing VSMC differentiation into osteoblast-like cells (reviewed in [103]). Interestingly, the work of Nielsen et al. revealed that galectin-3 has strong binding with glycoconjugate containing N-glycan. Perturbing glycoconjugates’ synthesis by interfering with various glycotransferases (enzymes that responsible for glycosylation process) decreases galectin-3 and glycoconjugates’ binding [101]. Finally, Ibarrola et al. demonstrated the beneficial effect of galectin-3 blockade in VC [105].

## 7. O-glycosylation and Vascular Calcification

Although O-glycosylation is a common glycosylation type, there are fewer studies that investigate it compared to N-glycosylation. O-GlcNAcylation is the process of the attachment of O-GlcNac to a target protein catalyzed by β-N-acetylglucosaminyltransferase (OGT) [80]. Heath et al. [79] was the first to report the importance of O-glycosylation (O-GlcNAcylation) and VC in a diabetes mellitus setting. Streptosozin-induced calcification mice showed an increase of aortic O-GlcNAcylation. Treatment with Thiamet-G, an inhibitor for O-GlcNAcase that is responsible for removing GlcNAcylation, shows more prominent calcification. Increasing GlcNAcylation through O-GlcNAcase knockdown promotes VSMC calcification by promoting RUNX2 upregulation and AKT enhancement. They found two O-GlcNAcylation sites of AKT, T430, and T479, that can promote its phosphorylation and therefore enhance VSMC calcification. A site-directed mutagenesis study revealed decreased O-GlcNAcylation and AKT phosphorylation. This disrupts the AKT-mTORC2 binding, which subsequently decreases RUNX2 activity and VSMC calcification.

A recent study reported O-GlcNAcylation of a YAP (Yes-Associated Protein) [106]. YAP is involved in vascular calcification through VSMC phenotype regulation [107]. Evidence was also provided by another group using endothelial cells. Uemura et al. [108] demonstrated the role of YAP/TAZ in development of VC by promoting intramembranous ossification via BMP pathways in endothelial cells. Interestingly, perturbation of O-GlcNAcylation by OGT knockdown counteracts high phosphate-induced vascular calcification in CKD through autophagy activation by downregulating YAP in VSMC [80]. The same research group by Xu et al. [109] also reported that glycosylation by OGT increases phosphate-induced vascular calcification in CKD through autophagy inhibition. Mechanistically, OGT increases KEAP1 glycosylation and subsequently ubiquitinates NRF2, increasing its degradation through autophagy. NRF2 has been shown to induce autophagy, and autophagy is involved in the amelioration of VC [110,111]. 

The vascular calcification process and its factors are similar to that of bone ossification [112] involving MV, mineral deposition and hydroxyapatite formation [15]. Accumulating evidence highlights the importance of phosphate in ossification. Fibroblast growth factor 23 (FGF23) was found to be the main phosphate regulator. It is synthesized by osteocytes and released into circulation [113]. They act with a co-receptor, klotho, in the kidneys to regulate phosphate [114]. FGF23 is known to cause hypophosphate familial tumoral calcinosis [115]. GWAS revealed GALNT3 as a top novel gene affecting bone mineral density and fracture risk [116]. GALNT3 is one of the enzymes responsible for O-glycosylation initiation. Recent studies reported that GALNT3 is the only GALNTs family member which initiates glycosylation of FGF23 [73]. The mechanism of FGF23 glycosylation by GALNT3 has been extensively studied by de las Rivas et al. [81]. GALNT3 specifically O-glycosylates FGF23 at Thr178. It seems that FGF23 has a role in VC. Epidemiological studies have shown the correlation of FGF23 and vascular calcification [117,118]. This is supported by molecular study [119], however, controversy exists [120]. Correa et al. [40] demonstrated expression of FGF23 in human calcified vascular tissue. Although FGF23 is known to cooperate with klotho in inducing VC, Jimbo et al. [39] demonstrated that FGF23 alone is sufficient to induce VC, induced by phosphate. Although there are currently no studies that directly correlate the O-glycosylation of FGF23 and VC, the insight from the molecular studies regarding FGF23 glycosylation and FGF23’s role in vascular calcification render correlation possible and warrant further study.

Most of all, there is some interesting evidence that the master regulator of osteoblast differentiation, RUNX2, is also subject to glycosylation by O-GlcNac modification [121]. RUNX2 is a transcription factor that is regulated by cytokines, growth factors, and hormones, including TGF-β, BMP, FGF, sonic hedgehog, vitamin D_3_, and estrogen [122]. Nagel and Ball [123] enhance the O-GlcNAcylation of RUNX2 by inhibiting OGA (an enzyme responsible for O-GlcNac removal). This increases the RUNX2 transcriptional activity induced by BMP2/7. Moreover, the increase in O-GlcNAcylation resulted in increased levels and activity of the bone formation marker ALP. Although there is currently no evidence that directly correlates RUNX2 glycosylation and VC, it has been well established that increased VSMC RUNX2 leads to increased vascular calcification [82,83]. Therefore, modification of its glycosylation might influence VC.

## 8. Proteoglycan and Vascular Calcification

Proteoglycan has been well-known for its role in vascular disease [124]. The response-to-retention theory stated that the extracellular matrix (proteoglycan), which has a negative charge, binds to positively charged LDL, and causes atherosclerosis [125,126]. For one of the proteoglycan, GAG, biosynthesis involves serial and a few of the glycosylation steps [62]. The initiation (which involves O-glycosylation and N-glycosylation) and the elongation (which involves other glycosylation types) make GAG one of the complex forms of glycosylation. GAGs found in mammals are chondroitin sulfate, dermatan sulfate, and heparan sulfate [62]. Accumulating evidence has reported that proteoglycan is involved in VC. 

Perlecan expression, one of heparan sulfate proteoglycan, is reported to decrease in aortic VC [84]. Purnomo et al. [85] demonstrated that GAG overproduction increases aortic calcification in murine CKD. They knocked-out EXTL2 to overexpress glycosaminoglycan and induced CKD by nephrectomy. They found that GAG overexpression induces VSMC’s differentiation to osteoblastic-like cells via a BMP2/smad1/5/8 pathway. Another proteoglycan, hyaluronan, has been reported to decrease VC [86]. Mechanistically, it decreases ALP and the expression of bone-related molecules including RUNX2, BMP2, and MSX2. Therefore, it inhibits VSMC to osteoblast differentiation. Interestingly, hyaluronan is also subject to complex glycosylation. Hyaluronan synthesis can be inhibited by 4-methylumbelliferone (4MU), which can inhibit UDP-glucuronosyltransferase (UGT) and also compete with GlcNAc and GlcUA as a substrate for UGT [127]. 

**Figure 1:**
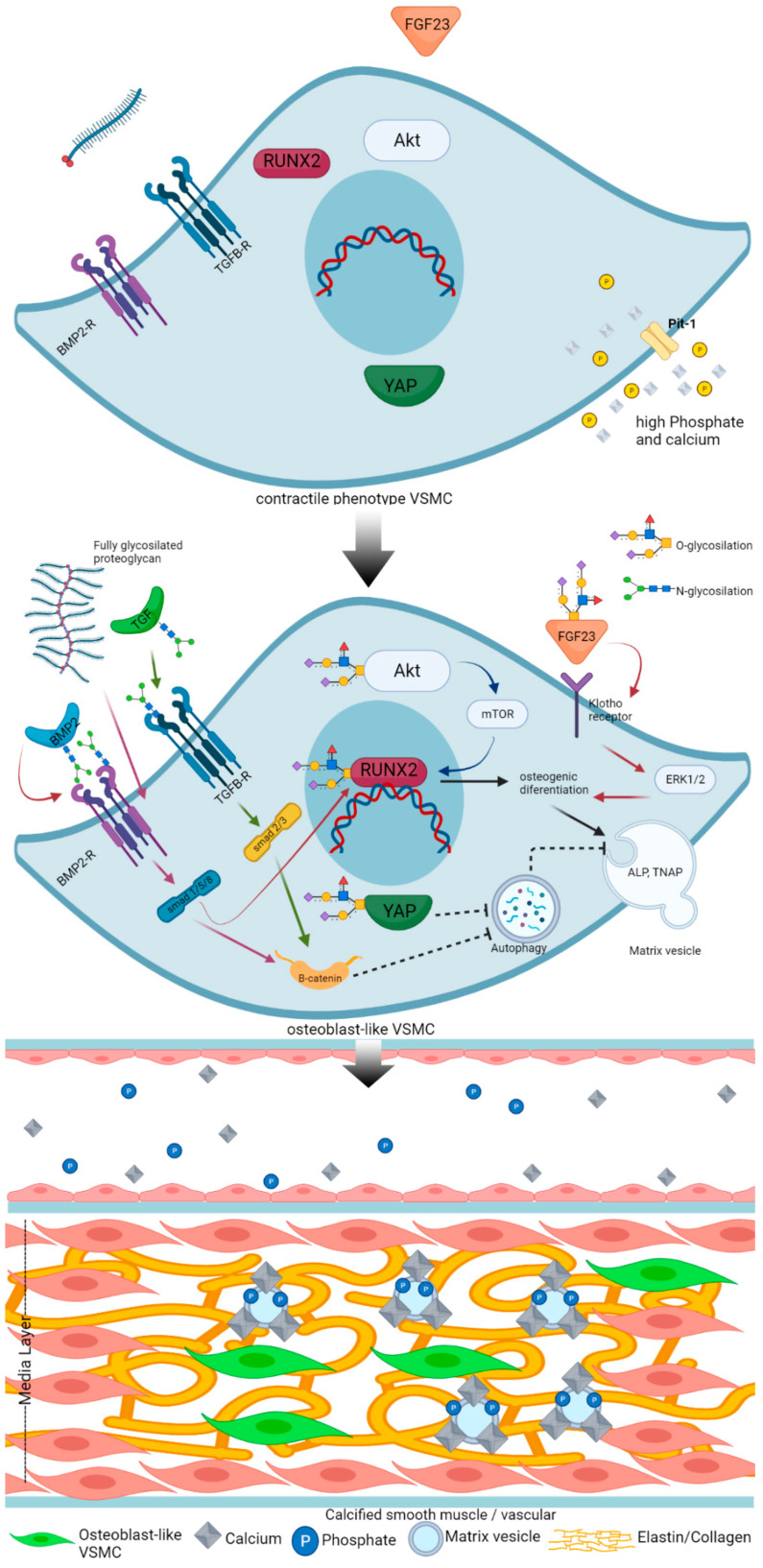
Proposed mechanism of how glycosylation of regulatory factors affects vascular calcification. Mechanism of vascular calcification involves loss of inhibitor and gain inducer. In the condition rich of phosphate and calcium, the VSMC transdifferentiates into osteoblast-like cell (**top** panel). Calcification inducers such as BMP2, FGF23, and ALP were recently reported to be modified by glycosylation. Even the master regulator of calcification, RUNX2, is also reported to be modified by glycosylation. The activation of an osteogenic program by RUNX2 subsequently induces formation and secretion of MVs. Secreted MVs contain enzymes that support calcification (e.g., ALP, (tissue nonspecific alkaline phosphatase (TNAP), which are potent calcification inducers). MVs serve as nidus for hydroxyapatite formation. Matrix vesicle formation is interfered by autophagy. Autophagy is considered to decrease vascular calcification. BMP2 increases calcification through inhibition of autophagy. BMP2 secretion is modified by glycosylation. Fully glycosylated proteoglycan induces VC via a BMP2-BMP2R-smad1/5/8 pathway. TGFβ-TGFβR induces calcification through smad2/3-β-catenin. This pathway inhibits autophagy, which eventually increases calcification. FGF23 secretion requires glycosylation by GALNT3. FGF23-klotho binding in VSMC activates an osteogenic program through ERK1/2. Overall, the increasing glycosylation will stabilize calcification inducers and eventually induce vascular calcification (**middle** and **bottom** panel). This figure is compiled from references [26,39,77,79,80,83,85,91,93,95,108,110,128,129,130,131]. The illustration was Created with BioRender.com.

Another member of proteoglycan, heparan sulfate, also showed its role in VC. A beautiful work by Borland et al. [87] demonstrated heparan sulfate in the extracellular domain of syndecan-4-regulating VSMC mineralization. Heparan sulfate biosynthesis involves several glycosylation enzymes, the most important being exostosin glycosyltransferase 1 (EXT1). Knocking down EXT1 increases VSMC mineralization. Mechanistically, the reduced heparan sulfate expression in syndecan 4’s extracellular domain causes a decrease in FGF2-induced AKT activation by decreasing FGF2-PKCα interaction. Therefore, heparan sulfate may be required to decrease VC. The mechanism of VC and how glycosylation affects it is illustrated in the Figure 1.

## 9. Conclusions and Perspective

The vascular calcification process resembles bone ossification, including the regulatory factors involved. The emerging evidence shows the importance of glycosylation in the development of disease. Numerous studies report the glycosylation of regulatory factors of VC and their various effects in VC. Here, we summarized the target of glycosylation in association with vascular calcification. This work will open future research regarding glycosylation and vascular calcification.

## Figures and Tables

**Table 1:** Glycosylation of proteins related to vascular calcification.

Enzyme	Glycosylation Type	Glycosylated Protein	Effect on VC	Reference
N/A	N-glycosylation	IGFR1	Decrease	[76]
N/A	N-glycosylation	BMP2, BMP2R	Increase	[77]
N/A	N-glycosylation	Fetuin-A	Decrease	
N/A	N-glycosylation	TGFβR	Increase	[78]
OGT	O-glycosylation (GlcNac)	AKT	Increase	[79]
OGT	O-glicosylation (GlcNac)	YAP	Increase	[80]
GALNT3	O-glycosylation (GalNac)	FGF23	Increase	[81]
OGT, OGA	O-glycosylation	Runx2	Increase	[82,83]
EXT1, EXTL2	Mixed	Proteoglycan	Various	[84,85,86,87]

Abbreviations: OGT: β-N-acetylglucosaminyltransferase; OGA: β-Nacetylglucosaminidase; GALNT3: pp-N-Acetylgalactosaminyltransferase 3; EXT1: Exostosin Glycosyltransferase 1; EXTL: exostosin-like glycosyltranferase 2; IGFR1: Insuline-like Growth Factor Receptor 1; BMP2: Bone Morphogenic Protein 2; BMP2R: Bone Morphogenic Protein Receptor; TGFβR: Transforming Growth Factor β Receptor; YAP: Yes-Associated Protein; FGF23: Fibroblast Growth Factor 23. GlcNac; N-Acetylglucosamine; GalNac: N-Acetylgalactosamine.
